# Esophageal regeneration following surgical implantation of a tissue engineered esophageal implant in a pediatric model

**DOI:** 10.1038/s41536-021-00200-9

**Published:** 2022-01-10

**Authors:** Sumati Sundaram, Todd Jensen, Tina Roffidal, Karissa Paquin, Heather Wanczyk, Michael D. Cockman, Shawyon Shadman, Christine Finck, William Fodor

**Affiliations:** 1 Biostage, 84 October Hill Rd., Holliston, MA 01746 USA; 2 Department of Pediatrics, University of Connecticut School of Medicine, 263 Farmington Avenue, Farmington, CT 06030 USA; 3 Department of Pediatric Surgery, CT Children’s Medical Center, 282 Washington Street, Hartford, CT 06106 USA; 4 Medical Metrics, Inc., Suite 300, 2121 Sage Rd., Houston, TX 77056 USA; 5 Madison Radiologists, 1221 John Q Hammons Dr., Madison, WI 53725 USA

**Keywords:** Regenerative medicine, Translational research, Mesenchymal stem cells, Tissue engineering

## Abstract

Diseases of the esophagus, damage of the esophagus due to injury or congenital defects during fetal esophageal development, i.e., esophageal atresia (EA), typically require surgical intervention to restore esophageal continuity. The development of tissue engineered tubular structures would improve the treatment options for these conditions by providing an alternative that is organ sparing and can be manufactured to fit the exact dimensions of the defect. An autologous tissue engineered Cellspan Esophageal Implant^TM^ (CEI) was surgically implanted into piglets that underwent surgical resection of the esophagus. Multiple survival time points, post-implantation, were analyzed histologically to understand the tissue architecture and time course of the regeneration process. In addition, we investigated CT imaging as an “in-life” monitoring protocol to assess tissue regeneration. We also utilized a clinically relevant animal management paradigm that was essential for long term survival. Following implantation, CT imaging revealed early tissue deposition and the formation of a contiguous tissue conduit. Endoscopic evaluation at multiple time points revealed complete epithelialization of the lumenal surface by day 90. Histologic evaluation at several necropsy time points, post-implantation, determined the time course of tissue regeneration and demonstrated that the tissue continues to remodel over the course of a 1-year survival time period, resulting in the development of esophageal structural features, including the mucosal epithelium, muscularis mucosae, lamina propria, as well as smooth muscle proliferation/migration initiating the formation of a laminated adventitia. Long term survival (1 year) demonstrated restoration of oral nutrition, normal animal growth and the overall safety of this treatment regimen.

## Introduction

The esophagus is a tubular organ that enables passage of food from the oral cavity to the stomach. The multi-laminated tissue structure has several functions that assist in the transport of nutrition to the stomach; (1) an epithelial mucosal lining of the luminal surface provides lubrication for transport of ingested food, (2) the mucosal lining also provides a protective immune barrier, preventing ingested microorganisms from infecting the underlying tissue, (3) innervated striated and smooth muscle layers within the adventitia stimulate peristalsis to forcibly push food down the length of the esophagus, and (4) the laminated structure provides a flexible muscular structure that can withstand the mechanical forces due to expansion and contraction from bolus ingestion^1^.

Several diseases of the esophagus, esophagitis, refractory chronic strictures, injury due to caustic burns, perforations, or other injuries can lead to end-stage organ dysfunction requiring surgical repair^2–10^. In addition, one of the most common congenital esophageal abnormalities is esophageal atresia (EA). Different forms of EA are classified based on the presence or absence of a fistula to the trachea (tracheoesophageal fistula TEF; Supplementary Fig. 1)^6^. EA occurs in approximately 1 in 4000 live births^10–16^, with Long Gap Esophageal Atresia (LGEA) accounting for 7–8% of all EA and is defined as a large gap in the developing esophagus resulting in two blind ends (Gross type A, Vogt type II; Supplementary Fig. 1)^17^. Surgery is required to repair LGEA and restore continuity of the esophagus to establish normal oral food intake. A variety of surgical approaches have been described such as delaying repair until the child grows, traction techniques, such as the Foker technique, to promote esophageal stretching, and the use of autologous donor tissues (i.e., intestine or stomach) to bridge the gap^17–23^. Position papers delineating optimal surgical repair for LGEA by both the International Network of Esophageal Atresia (INoEA) and the American Pediatric Surgery Association (APSA) advocate that native esophagus is best to use whenever possible^23,24^. Unfortunately, delaying repair until the child grows or the use of the Foker or traction techniques have significant costs, complication rates, lengthy hospital stays, and significant morbidities^25–30^. Therefore, the development of novel approaches that bridge a primary long gap, repair a failed EA treatment^9,10^ or repair segmental lesions due to injury or disease, while synchronously preserving the native esophageal tissue, are highly desired and is the focus of this research.

The Cellspan Esophageal Implant^TM^ (CEI) is a combination product composed of autologous adipose derived mesenchymal stromal cells (Ad-MSC) seeded onto a polyurethane tubular mesh cell delivery device (Cellframe^TM^ Technology). The CEI is designed to bridge the gap in LGEA, stimulate neo-esophageal tissue regeneration and preserve the native esophagus. Previous studies utilizing the CEI in adult models of esophageal reconstruction demonstrated the formation of neo-tissue spanning the entire length of the implant, restoring continuity of the esophagus with vascularized, viable host tissue^31,32^. In these pre-clinical porcine studies, a large portion of native esophagus was resected and replaced with the CEI, using an end-end anastomosis (Supplementary Fig. 2)^31,32^. At 21 days post-implantation, the scaffold component which failed to integrate into the developing tissue was endoscopically removed, revealing a continuous, regenerated, fibrovascular tube of tissue^31,32^. Endoscopic evaluation of the lumen revealed the development of a mucosal epithelial layer across the implant that was fully formed by 90 days post-implant^32^. Functionally the animals were able to eat and gain weight^31,32^. Macroscopic and microscopic histologic analyses at multiple necropsy time points post-surgery revealed the early formation of fibrovascular neo-tissue followed by epithelialization of the lumenal surface. Nine-month post-surgical endoscopy analyses also demonstrated an intact epithelialized lumen^32^. A similar strategy for esophageal reconstruction was tested in a porcine model, where autologous bone marrow MSCs (BM-MSC) were seeded onto an acellular small intestine submucosal construct. The results from this study demonstrated re-epithelialization as well as the development of muscular structures in animals surviving beyond 50 days post-implant with the longest survival time of 119 days^33^. However, this study repaired the abdominal esophagus and used 2 surgical procedures, an initial surgery to “maturate” the construct in the omentum and then a second surgery to repair a 3 cm full circumferential resection with the maturated tissue engineered tube^33^. The 2 surgical procedures and the ‘maturation process’ of the graft may prove difficult to translate this procedure to a clinical setting.

The first in human use of a clinical grade tissue engineered construct, utilized a GMP manufactured CEI and demonstrated successful implantation into a patient that required a full circumferential segmental esophageal resection to remove a large chest tumor encroaching on the esophagus^34^. Similar to the animal data, the CEI functioned to stimulate tissue regeneration and restored esophageal continuity with a living vascularized, epithelialized biologic conduit up to 7.5 months post-implantation. These pre-clinical animal studies and the successful translation of the technology to a human patient demonstrates the feasibility of utilizing this technology for segmental esophageal reconstruction. To support the use of the CEI for LGEA as a primary treatment or to repair a failed primary treatment, Jensen et. al. demonstrated that Ad-MSCs can be isolated from LGEA patients and that the cells proliferate, maintain a MSC phenotype and can be used to produce a CEI^35^. The current study utilizes pediatric sized implants, expands on the tissue analysis time course for up to 1-year post-implantation (vs. 2.5 months^32^), includes surgical controls, computed tomography (CT) scan analyses of the developing tissue and employs more comprehensive clinical management procedures. Therefore, the goal of this study was to gain a better understanding of the time course of the regenerative process following implantation of a pediatric-sized CEI in a porcine pediatric esophageal reconstruction model. In addition, the evaluation of tissue deposition using CT, animal health, growth, development and overall long-term safety using clinically appropriate management procedures over the course of the 1-year survival time point establishes a clinical paradigm for the CEI.

## Results

### pAd-MSC expansion and characterization

pAd-MSCs had an average population doubling time of 22.0 h ± 2.2 h, an average viability of 89.8% ± 6.9%, determined by trypan blue, and expected cell morphology with plastic adherence (Supplementary Fig. 3a). Flow cytometry was performed with multiple MSC cell surface markers, as well as negative controls to characterize the cells. MSCs were characterized as CD44, CD73, CD90, CD105, CD271, and CD146 positive, and CD14, CD45, and SLADRII negative (Supplementary Fig. 3b, c).

### CEI Seeding and characterization

Ad-MSC cultures were expanded in two-dimensional flasks, harvested and seeded onto the scaffold in a custom rotating bioreactor. Glucose and lactate levels from the expended media were analyzed to measure cell metabolism. Fresh medium without cells contained on average 95 mmol/L of glucose and 4 mmol/L of lactate, which is indicated by the starting points on the metabolism graph in Supplementary Fig. 5b. The 6-day expended media from all CEI’s had an average glucose concentration below 70 mg/dL and an average lactate concentration greater than 9 mmol/ L (Supplementary Fig. 5b), indicating that the cells were metabolically active throughout the CEI production period. Biopsy punches were taken from along the length of the quality control CEIs (QC) and tested for cell viability using the LIVE/DEAD assay (Supplementary Fig. 5c). All samples had scores of 4/4, indicated an abundance of live cells and few dead cells in all 4 quadrants of the CEI punches (Supplementary Fig. 5C). All QC samples showed cells penetrating through at least 25% of the scaffold material (Supplementary Fig. 5d). All QC CEIs displayed cell counts greater than 4,000 cells / mm^2^, indicating viability and growth of cells on the scaffold during incubation (Supplementary Fig. 5e). Finally, conditioned media taken from the bioreactors at harvest were analyzed for the presence of specific growth factors and cytokines including VEGF-A, IL-6, IL-8, and total MMP-2 (Supplementary Fig. 5f). Overall, all the manufactured CEIs passed the quality control specifications for in vivo implantation based on metabolic activity, DNA content, LIVE/DEAD, and scaffold penetration.

### Implantation surgery

Twelve animals were implanted and recovered from surgery. Two early terminations took place in the 90-day cohort. One animal was euthanized one day after surgery due to hindlimb paralysis and another animal was euthanized 12 days after surgery due to pericardial effusion and lung disease (Table 1). Autopsy pathology indicated that the hindlimb paralysis was not caused by surgery and the lung disease appeared to be present well before surgery. The other early termination was in the 365-day cohort at Day 297 due to dehydration, vomiting, and decreased body condition associated with an incidental small intestine volvulus (Table 1). Overall, the use of this Ad-MSC seeded CEI graft was safe and feasible.

**Table 1:** Overall study summary.

Study duration (Days)	Cohort design animal numbers per group		Survival to term		Early deaths	
	Test	Surgical Control	Test	Control	Test	Control
Cohort 3 30 ± 3 days	4	1	4	1	0	0
Cohort 2 90 ± 3 days	5	1	3	1	1 @ D1 1 @ D12	0
Cohort 1 365 ± 15 days	3	1	2	1	1@D297	0

Study design matrix indicating study duration (cohort), numbers of animals per cohort and survival times for test (AD-MSC seeded scaffold implanted) and control animals (surgical esophagectomy with primary anastomosis).

*D1* day 1, *D12* day 12, *D 297* day 297.

### Endoscopy studies and stent exchanges

**Fig. 1:**
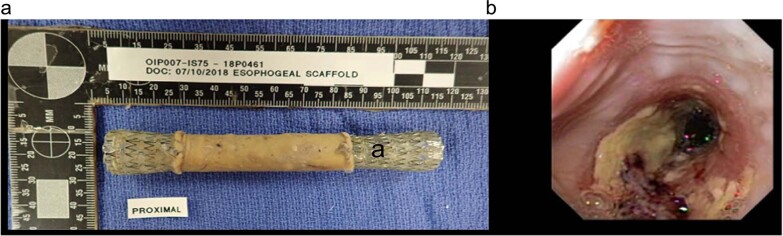
Retrieval of scaffold and initial stent followed by an esophagram at 21 days post implant. At the time of the first endoscopy (21 days post-surgery), the initial stent with the scaffold attached is removed (**a**). An endoscopic image illustrating the lumen of a patent fibrovascular tube of tissue following scaffold and stent removal (**b**); white arrow indicates regeneration zone.

**Fig. 2:**
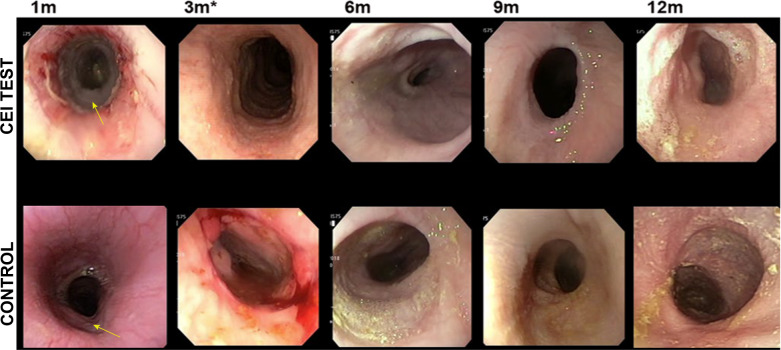
Endoscopic evaluation of the implant at multiple time points. Endoscopy images taken at 1 month (all animals), 3 months (90 day and 365-day cohorts), and then sequentially at up to 12 months (365-day cohort). CEI implant recipients (**a**); surgical control cohort (**b**). The images illustrate that at 1 month (arrow) there is still a regenerating area in the test animal. By 3 months (asterisk; control and test animals) the epithelium appears to be regenerated as evident by the smooth glossy surface that persisted up to the 12 month time point in the long term cohort (365 day).

In all surgical control and CEI test animals, the initial stent was removed endoscopically at day 21 post surgery. In the CEI recipients, the scaffold component of the CEI was also removed as it adhered to the stent and released from the neo-tissue (Fig. 1a). Endoscopic visualization of the newly formed adventitial tissue revealed a complete contiguous tube that spanned the implant site. The tissue at the implant site appeared red and un-epithelialized (Fig. 1b). All animals were re-stented and returned to their pens. The 30-day cohort was euthanized at 9 days post re-stenting and underwent necropsy analyses revealing an intact tubular structure with un-epithelialized lumen in both the surgical control group and in the CEI cohort (Figs. 5a, d and 6a, b). Epithelialization was complete by 3 months post-implantation in the both the CEI implant and the control groups as determined by endoscopy, gross histology, and MT histology (Figs. 2a, b, 5b–f and 6c–f, respectively). Stents were utilized to keep the lumen patent and on average were removed by day 120 in the 365-day cohort. Stents in the 30-day and 90-day cohorts were utilized throughout the in-life period. There were no stent migrations noted in the 30-day cohort, 2 migrations noted in the 90-day cohort (surgical control animal) and 16 migrations noted in the 365 cohort (6 in the surgical control animal and 10 in the 3 CEI test animals; Table 2). Cohort 1 animals (365 ± 15 day; control and test animals) underwent several balloon dilations post final stent removal to treat esophageal strictures over the course of the study. (Table 2).

**Table 2:** Summary of endoscopic interventions.

Animal ID	Survival Day	Test/Control	Endoscopic tissue evaluation	Stent interventions		Balloon dilations^a^		Number days w/o dilation (prior to termination)
			Mucosa complete (POD)^2^	Permanent stent Removal (POD)	Total stent Migrations	# During Stent period	# Post Stent Removal	
*30-Day Cohort (+/− 3 days)*								
18P0973 (F)	28	Test	N/A^3^	N/A	0	0	N/A	28
18P0974 (F)	27	Test	N/A	N/A	0	0	N/A	27
18P0637 (F)	29	Test	N/A	N/A	0	0	N/A	29
18P0638 (F)	29	Test	N/A	N/A	0	0	N/A	29
18P0976 (M)	27	Surgical control	N/A	N/A	0	0	N/A	27
9*0-Day Cohort (+/− 3 days)*								
18P0633 (M)	90	Test	90	N/A	0	0	N/A	90
18P0634 (M)	91	Test	91	N/A	0	0	N/A	91
18P0975 (M)	90	Test	90	N/A	0	0	N/A	90
18P0636 (M)	92	Surgical control	N/A	N/A	2 distal	0	N/A	92
*365-Day Cohort (+/− 15 days)*								
18P0459 (F)	297	Test	63	127	4 distal	1	5	105
18P0460 (M)	349	Test	62	117	2 distal	1	10	76
18P0461 (M)	350	Test	63	141	4 distal	2	11	20
18P0458 (F)	350	Surgical control	N/A	98	6 distal	6	4	197

*POD* Post-Op Day, *N/A* not applicable.

^**a**^It was rare that a dilation would need to be performed when the stent was in place. If it were difficult to place the stent past a stricture, then a balloon dilation was performed before stent deployment. After permanent removal of the stents in cohort 1 (365-day group), monthly endoscopy and barium swallow procedures were performed to evaluate the esophagus implant region. If the presence of a stricture greater than 10% of total diameter of proximal esophagus, a balloon dilation was performed.

### Evaluation of tissue regeneration via computed tomography (CT) scan

**Fig. 3:**
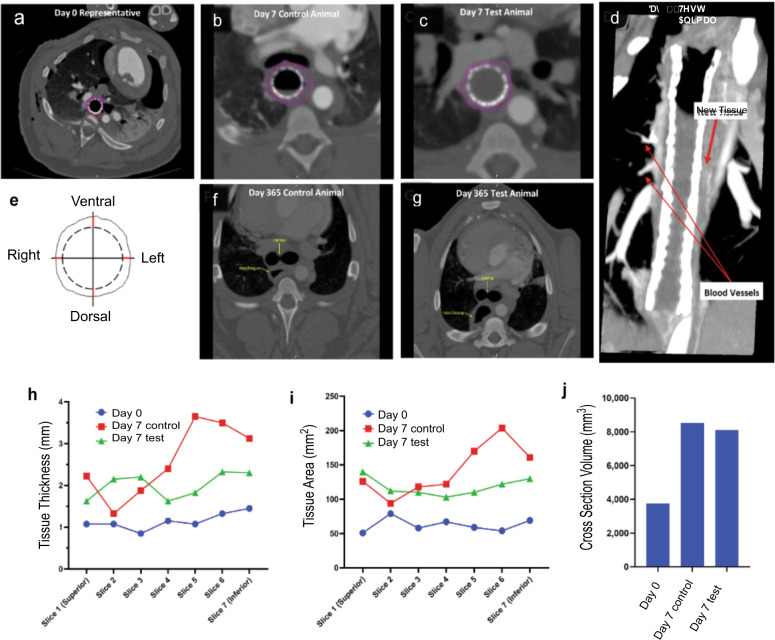
Analysis of esophageal tissue formation using computed tomography imaging. Test and surgical control animals were evaluated by CT Scan at various timepoints throughout the study with and without the addition of contrast dye (**a**–**d, f**, **g**). Vessels sprouting from the aorta to the newly formed tissue were observed at Day 21 via CT Scan with contrast (**d**). Cross-section images were analyzed by Medical Metrics Inc. (Houston, TX) for tissue thickness (mm), tissue area (mm^2^) and tissue volume (mm^3^). The averages of each timepoint for each slice was graphed below (**h**–**j**). It’s important to note that the control tissue was a slightly larger area compared to the regenerated tissue area.

CT images at Day 0 demonstrated that the area near the carina was within the implant zone as indicated by the presence of the esophageal stent, with an absence of tissue surrounding it (Fig. 3a). CT images obtained at Day 7 indicated that tissue was forming within the implant zone in both the control and test animals (Fig. 3b, c). Reformatting of the CT images in the coronal plane demonstrated new tissue formation with blood vessels at day 21 in test animals (Fig. 3d). Tissue thickness was measured at seven different slices and each slice was measured in four distinct places: ventral, left, posterior, right (Fig. 3e). The esophageal tissue in the 365-day cohort appeared to be similar in both the CEI group and the surgical control (Figs. 3f, g). However, it was difficult to distinguish the neo-tissue vs. the native tissue. Day 7 results were utilized to determine how the tissue was growing in comparison to control tissue. The day 7 results detected tissue formation circumferentially along the length of the CEI implant zone. Tissue thickness measurements, approximating the center of the implant zone in the test animals and the anastomotic site in the surgical control, were comparable (Fig. 3h, i; slices 1–7). Volumetric measurements from several points through the implant zones also showed comparable tissue volume in the CEI recipients as compared to the controls (Fig. 3j).

### Evaluation of tissue function via esophagram

**Fig. 4:**
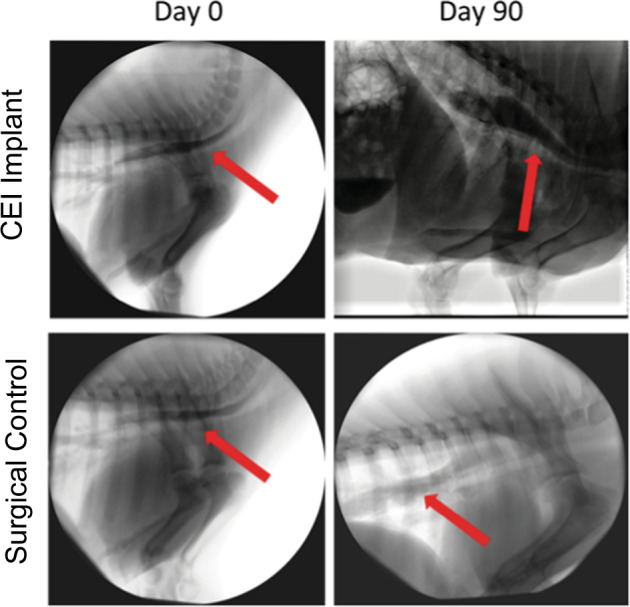
Representative barium swallow esophagram images from a CEI recipient and a surgical control animal. Esophagram analysis following a barium swallow test from Control and CEI Test animals at 30- and 90 days post surgery (red arrows indicate barium in esophagus).

Video from the fluoroscopic studies performed at 30 and 90 days demonstrated a patent lumen without extravasation of barium. All barium (red arrows) traversed to the stomach in a timely fashion (Fig. 4).

### Gross histology

**Fig. 5:**
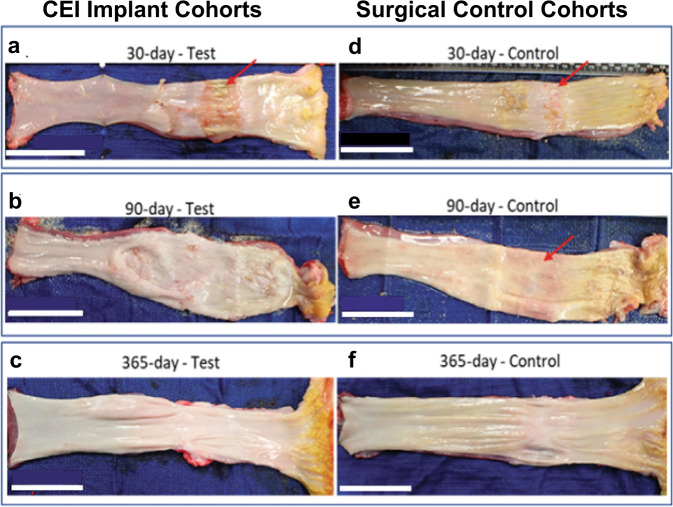
Gross histology of esophageal tissue from 30-, 90- and 365-Day cohorts. Representative gross images of explanted esophagus containing treatment site at necropsy. Esophagus from test animals (treated with the CEI) and control animals survived to various times: 30-day survival (**a**, **d**; arrows indicate area that is not epithelialized); 90-day survival (**b**, **e**; arrow indicates area that is not epithelialized); 365-day survival (**c**, **f**). White scale bars = 5 cm.

Gross histology revealed that at 30 day, the test animal had a larger area that was not epithelialized (red arrows) compared to the control at 30 days (Figs. 5a, d). At 90 days, there was complete epithelialization in both groups (Fig. 5b, e). At 365 days, the test and control gross histology were strikingly similar (Fig. 5c, f).

### Histologic analyses

**Fig. 6:**
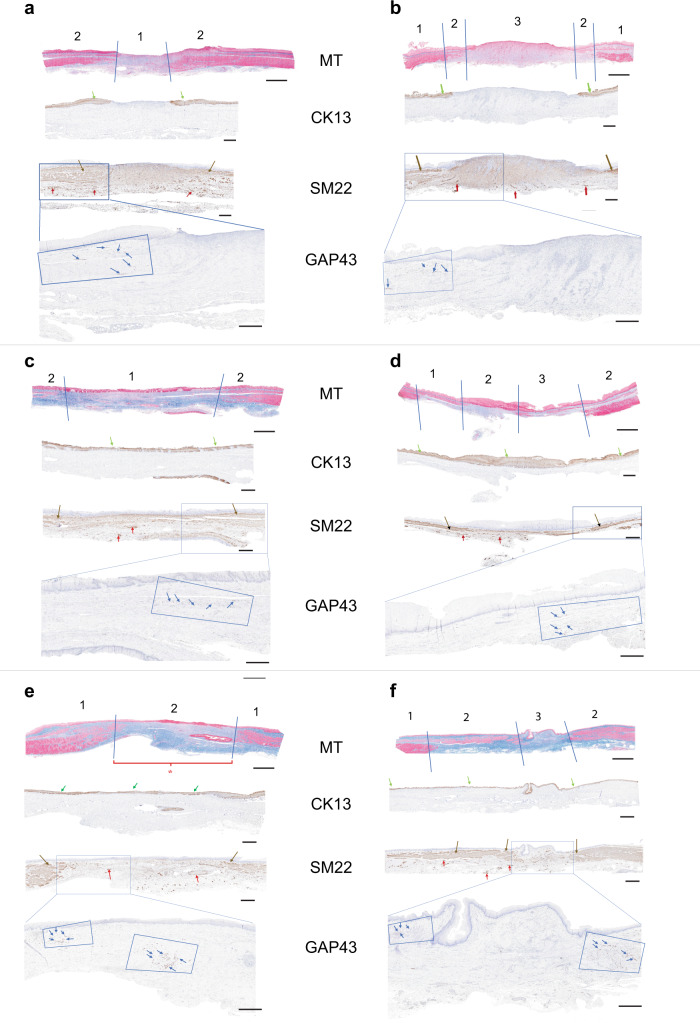
Mason’s Trichrome stain and immunohistochemical analysis of explanted esophageal tissue. Mason’s Trichrome (MT) stain and Immunohistochemical analysis (cytokeratine-13, CK13; smooth muscle transgelin, SM22; and growth associated protein-43, GAP43) of excised esophagus from the 3 surgical control animals (CNTL: **a** 30-day; **c** 90-day; **e** 365-day) and the CEI implant recipients (**b** 30-day; **d** 90-day; **f** 365-day). The histologic stains are indicated in the center of the tissue samples next to each micrograph. Scale bars in the MT = 4 mm. Scale bars in the CK13, SM22, and GAP43 panels = 2 mm. **a**–**f** Top panel, MT-stained tissue illustrating the resected area of the surgical controls (CNTL) and the implant regions of the CEI recipient animals at 30-days, 90-days, and 365-days post-surgery. Tissue Zones are labeled as 1 (native tissue flanking the resection) and 2, the resected area in the controls (**a, c, e**) and Zone 1 (native), Zone 2 (transition zone) and Zone 3 (central fibrovascular tissue) in the CEI recipients (**b, d, f**). Scale bars are indicated in each panel. MT scale bar = 4 mm; scale bar for CK13, SM22, and GAP43 panels = 2 mm. **a**–**f** IHC. Cytokeratin (CK13) immunohistochemistry (IHC) illustrates the cytokeratin positive epithelial layer spanning the resection (green arrows) in the CEI implant. SM22 IHC identifies the smooth muscle components of the native tissue and in the regenerating regions in the CEI implant recipients (brown arrows). SM22 also identifies the vascular structures throughout the resected area and within the CEI implant zones (red arrows). GAP43 IHC identified growth cone positive neuronal structures at the border of the resected area and at the proliferative fronts of the regenerating smooth muscle tissue migrating into the CEI implant zones (blue arrows with the blue boxes). Boxed region in the SM22 IHC panel represents the area in the GAP43 IHC panels. **e** The red bracket labeled with an asterisk indicates the area of the resection in the surgical control that exhibits persistent non-regenerating fibrovascular tissue across zone 2 at 1-year post-surgery.

**Fig. 7:**
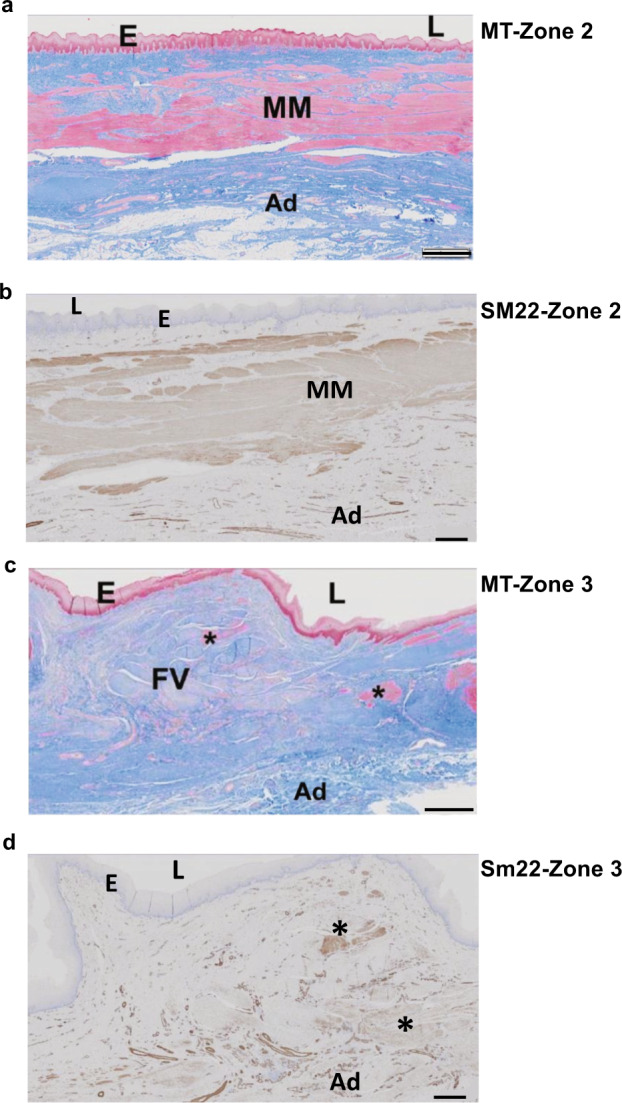
Tissue analysis of 365-day CEI recipient. High powered microscopic histologic analysis of the 365-day CEI recipient. Panels **a**–**d** are high powered images of the CEI implants taken from Zone 2 (**a**, **b**) and Zone 3 (**c**, **d**) from the 365-day recipient (**a**, **c** = MT stain; **b**, **d** = SM22 stain; E = epithelium; L = lumen; FV = fibrovascular tissue; Ad = adventitia; asterisks indicate areas of muscle cell staining in **c**, **d**). Scale bars = 1 mm in the MT stained slides (**a, c**) and 500 μm in the SM22 stained slides (**b, d**).

To evaluate tissue regeneration and healing across the implantation site, the tissue obtained at necropsy was labeled and divided into Zones as illustrated in the Mason Trichrome (MT) stained tissue (Fig. 6a–f) and further described in Supplementary Fig. 7. Mason’s Trichrome (MT) histology and immunohistochemical analyses (IHC) were performed to determine the structure and phenotype of the regenerating tissue and identify specific cellular protein expression patterns. The analyses included the identification of epithelial, smooth muscle, vascular and neuronal cellular proteins. Cytokeratin-13 (CK13) identified the regeneration of the luminal epithelium and determined the time course of epithelial regeneration in both the control and test group (Fig. 6a–f). Transgelin (SM22) was used to identify smooth muscle cell components of the tissue in the adventitia as well as in neo-vasculature structures (Figs. 6a–f, 7a–d). The presence of GAP43 positive cells within the newly formed tissue identified neuronal growth cone regeneration and/or neuronal cell proliferation that appeared to be associated with smooth muscle proliferative fronts (Fig. 6b, d, f) in the CEI recipient animals. In the control animals, neuronal cell proliferation may also be associated with nerve regrowth that occurs after the segmental esophagectomy and anastomosis (Fig. 6a, c, e).

MT histology of the 30-day cohort demonstrated fibrovascular tissue across the anastomotic site in the control (Zone 2) and across the CEI implant from Zone 1 through Zone 3 (Fig. 6a, b). The MT histology and CK13 IHC revealed an absence of a mucosal epithelial cell layer, as well as an absence of the laminated structure of the esophageal adventitia, i.e., the absence of both smooth and striated muscle tissue (absence of SM22 staining in zone 2, Fig. 6a, b). MT histology of the 90-day cohort demonstrated a complete restoration of the luminal epithelium in both the control group and in the CEI implant recipient (Fig. 6c, d, MT and CK13 IHC). In addition, SM22 IHC demonstrated the presence of smooth muscle tissue within the CEI implant region spanning from Zone 1 into Zone 2 (Fig. 6d, SM22 panel), whereas, in the surgical control group, the fibrovascular scar persisted with no evidence of smooth muscle proliferation (Zone 2, Fig. 6c). Interestingly, the histologic findings in the control animal at day 365 post surgery demonstrated that the loss of muscle integrity in Zone 2 did not resolve by 365 days (Fig. 6e). However, the MT and CK13 staining on the tissue from the 365-day CEI test group demonstrated a complete epithelium as well as clear evidence of a regenerating adventitia, as determined from the presence of smooth muscle tissue extending through Zone 2 and the development/regeneration of the muscularis mucosae (Figs. 6f, 7a–d, Supplementary Table 6). In addition, the identification of vascular structures and smooth muscle cell tissue in Zone 3 suggests that the tissue was continuing to remodel (Fig. 7c, d).

Standard histopathology was also performed on tissues obtained from the pancreas, stomach, gallbladder, liver, kidneys, heart, ovaries, lymph nodes, brain, and pituitary glands from all test and control animals. Polymorphonuclear cell infiltrates, sinus histiocytosis, edema/fibrin and multinucleated giant cells were observed in the mediastinal and hepatic lymph nodes, reflecting ongoing or resolved changes in the upstream organs drained by these lymph nodes (esophagus, lungs, heart, and liver). No abnormal incidental pathology findings were observed from any of the other tissues (data not shown), from the CEI recipients, indicating that the CEI product is safe and well tolerated.

## Discussion

Current surgical treatments for the repair of the esophagus due to disease, injury or congenital defects, including long gap esophageal atresia (LGEA) is laden with significant morbidity. The feasibility of an innovative surgical option that utilizes an autologous cell seeded removable scaffold was demonstrated in a large animal model^31,32^ and in an Expanded Use Clinical single case study^34^. The goal of this study was to apply this technology to a pediatric porcine model to: (1) understand the time course of the regeneration process in young animals; (2) demonstrate that the regenerated tissue leads to the restoration of esophageal function; and (3) demonstrate that the CEI is safe and well tolerated in young animals, which is essential for clinical translation into children.

All CEIs met quality control metrics prior to release for implantation (Supplementary Fig. 5). The conditioned media from the cell-seeded scaffolds used in this study consistently demonstrated high levels of key angiogenic cytokines and growth factors critical for wound healing and vascularization of the graft (VEGF-A, IL-8, and MMP-2, Supplementary Fig. 4f). The stimulation of angiogenesis is a well-documented paracrine function attributed to mesenchymal stromal cells (MSCs)^36–38^ and is hypothesized to be a critical functional feature of the CEI mechanism of action (MOA). The effect of these cytokines and growth factors most likely contributes to the neovascularization of the regenerating tissue, preventing necrosis and establishing a pro-growth platform for the regenerating tissue. Previous large animal GLP studies addressing acute toxicity and biodistribution demonstrated the persistence of GFP labeled Ad-MSC for 3 weeks post implant and only identified GFP labeled “Ad-MSC pericytes” associated with neovascular structures and not contributing to the bulk of the neo-tissue (WF personal communication, Biostage, Inc., Approved IND 19223).

A unique aspect of this technology is that the scaffold component of the CEI serves as a temporary cell delivery device that does not integrate into the developing neo-tissue and can be retrieved endoscopically at 21 days post-implantation (Fig. 1), leaving behind autologous tissue that progressively regenerates the tubular organ. The newly regenerated tissue was able to bridge a 5 cm esophageal gap, restoring the lumenal epithelia by 3 months with continued active regeneration of the laminated adventitia over the course of the 1 year follow up time point.

An essential component for clinical translation is establishing clinically relevant animal management procedures (post-surgery recovery, G-tube feeding, endoscopic evaluations, stent, and stricture management) that mimic the management of a neonatal long gap esophageal atresia patient following surgical intervention. Clinical management for long gap esophageal atresia typically requires repeated endoscopy and dilation. The use of a stent is not routine but was essential in this model to keep the fibrovascular tissue of the regenerating esophagus open for the first 4–6 months. A similar approach was used in the BM-MSC/SIS model^33^, where stents were required to maintain patency and prevent stricture formation. Interestingly, stent migration was a major cause for animal morbidity and early euthanasia^33^. Therefore, stent management was an essential component of the animal care in this study and included routine stent exchanges and the treatment of strictures with pneumatic balloon dilation in the long-term survival group. As delineated in Table 2, management of stents and strictures was a dynamic process requiring continual monitoring and intervention. In the 365-day cohort, there were 16 stent migrations and 40 balloon dilations. Compared to the clinical scenario, this is more than the typical clinical case, however, it is not unexpected given the time course for regenerating a tubular segment of tissue from a tissue engineered cellular construct. We also believe that it is a limitation of the animal model, i.e., the use of human stents in the pig^33^. To mitigate stent migration that was observed in cohort 1 (365-day animals), the diet of Cohort 2 (90-day) and Cohort 3 (30-day) was modified, such that they were not transitioned to oral solid food post scaffold removal (Day 21) and while stent usage was still indicated. Therefore, the 90-day animals maintained a liquid to semi-liquid oral diet until termination and the 30-day animals maintained tube feedings. This appears to have mitigated the risk of stent migration, as only 2 stent migrations were observed in Cohort 2 (90-day, surgical control animal) and zero migrations in cohort 1 (Table 2).

A component of the “in-life” monitoring included tracking the growth and weight of the animals. The feeding regimen utilized in this study allowed for tissue healing in the first 21 days without oral food. This approach is very similar to the standard of care for neonatal patients after an esophageal atresia repair^39^. The functional performance of the regenerated esophagus was evident in the growth curve where animal weights of the control and test animals were comparable through 6 months post-surgery (Supplementary Fig. 6). Although the control animal gained approximately 20% more weight than the test animals, the test animals gained weight steadily over the course of the study and eventually tripled their size. In the clinical scenario, surgery can slow the growth of a neonate, so this finding was not surprising. Esophagrams were performed by feeding the piglets barium mixed with food during live fluoroscopy (Fig. 4). At 30 and 90 days, the bolus of liquid moved steadily into the stomach without leak but with some reflux in the test group. This is an important finding, as the tissue that regenerated does provide a conduit with motility, however, detection of active peristalsis using manometry was not performed and will be investigated in future studies.

In order to track the in vivo tissue development and healing process without intervention, we applied CT imaging to monitor tissue growth and regeneration across the implant during the first 21 days, as well as determined the changes in tissue thickness over the course of the study. The ability to demonstrate tissue development in the regenerating zone that circumferentially bridges the gap between the esophageal ends and is thick enough to support removal of the stent and scaffold is imperative for clinical translation. The neo-adventitial tissue deposition and regeneration occurred circumferentially along the entire length of the implant zone (Fig. 3). Mechanical strength of the regenerated tissue increased over time and at 365 days was similar to native tissue (data not shown, W.F. personal communication; manuscript in preparation). This is a significant feature important to clinical translation to ensure that the regenerating tissue can maintain tissue integrity following a bolus of food with passage to the stomach without rupture.

The histologic analysis at 30-days supported the CT imaging results and demonstrated a complete circumferential regenerative process with contiguous tissue spanning the gap between the native ends of the esophagus. At the 90-day time point, the neo-tissue is completely epithelialized. Neovascularization occurred in the developing tissue and was evident in the 30-day tissue analysis and persisted in both the 90 and 365-day analyses (Supplementary Tables 3–6). Neo-vascular structures were present throughout the implant zones of all cohorts, supplying the implant zone with nutrients and oxygen and to prevent necrosis. Immunohistochemistry demonstrated that muscle and nerve cells were present in Zone 2 in the 90-day test cohort that became more pronounced and abundant in Zone 2 in the 365-day test cohort (Fig. 6d, f) indicating that the tissue was regenerating and developing into a’laminated’ structure, approaching a structure similar to the native esophageal tissue. The regenerated tissue looked most similar to that of normal esophagus at the 365-day time point, however, it is important to note that muscle layers were not complete at 365 days, which could be critical for complete peristalsis through the implant zones. Longer term studies >1 year will be required to see if the tissue becomes completely laminated with a structure comparable to native esophagus. An interesting and important observation in the surgical control animals was that the adventitial region surrounding the anastomotic site never completely repaired itself. Instead, a gap in the tunica muscularis was evident even by day 365. This finding is most likely due to ‘growth inhibitory’ collagen deposition and scar formation, however, further studies will be required to understand this phenomenon. This is in contrast to the CEI recipients, where the tissue continues to remodel towards a phenotype and structure similar to native tissue (Fig. 6f, g, h). The time course features of the regenerating tissue in the CEI recipients progresses from (1) fibrovascular tissue to (2) epithelialized fibrovascular tissue to (3) tissue with laminated features, including the sub-epithelial lamina propria and muscularis mucosae, as well as adventitial muscular components with evidence of neural growth cones at the leading edges of smooth muscle proliferation (Fig. 6; Supplementary Tables 3–6).

In conclusion, this study reports on the longest (365 ± 15 days) pediatric porcine animal study of esophageal reconstruction using a synthetic scaffold seeded with autologous mesenchymal stromal cells to stimulate esophageal regeneration. We demonstrated feasibility of this treatment modality for segmental repair of the esophagus and as a model for pediatric long gap esophageal atresia repair. This study established that a segment of the esophagus can be regenerated given an appropriate wound healing platform. Further investigation into the molecular mechanisms of the wound healing and regenerative processes are warranted and could yield important information for refining this technology. The adherence to clinical management principles clearly sets the stage for its translational application to patients requiring esophageal repair.

## Methods

### Pre-clinical animal study design

This study enrolled 15 Yucatan minipigs ranging from 40 to 50 days of age that were assigned to three cohorts with different survival time points (Cohort 1; 365 ± 15 days, Cohort 2; 90 ± 3 days and Cohort 3; 30 ± 3 days post-implantation. American Preclinical Sciences, Minneapolis, MN IACUC protocols OIP007-IS75/OIP010-IS75). The number of animals, time points, and groups are described in Table 1. All animals enrolled in this study passed a physical exam including routine bloodwork with values that were within normal range. A surgical control animal was included for each survival time point. All surgical control animals underwent a 2 cm esophageal resection followed by a single anastomosis to reconnect the proximal and distal ends of the esophagus. All test animals underwent a 5 cm segmental esophagectomy followed by the implantation of the CEI to bridge the gap between the proximal and distal esophagus using end-to-end anastomoses.

### MSC isolation and expansion

Adipose tissue (6–12 grams) was harvested from the midline pelvic region from all animals at ~28 days prior to implantation. Adipose tissue biopsies were shipped overnight at 2–8 °C in αMEM (Gibco, Grand Island, NY) + 0.1% gentamicin at 2–8 °C with temperature monitoring to the laboratory. Ad-MSCs were isolated from adipose as described^32^ Cells were plated at a density of 40 mg (of pre-digested tissue)/cm^2^ in either T75 or T150 tissue culture treated flasks in StemXVivo culture media (R&D Systems, Minneapolis, MN). Medium was refreshed every 48 h until they reached 70–80% confluence. Cells were cultured in 2-D format and then seeded at a density of 1143 cells/cm^2^ onto each scaffold.

### Flow cytometry

MSCs were characterized via flow cytometry as previously described at the beginning of the second passage^32^. Briefly, cells were harvested and washed two times with wash buffer (DPBS (Ca^-^/Mg^-^) (Gibco, Grand Island, NY), 1% bovine serum albumin (Gibco, Grand Island, NY) and aliquoted into tubes containing 1 × 10^5^ cells in 100 µL wash buffer. Primary antibodies were added to the aliquots according to Supplementary Table 1 and incubated for 30 min in the dark on ice. Tubes were washed twice with wash buffer and centrifuged at 1000 rpm for 2 min at 4 °C. Flow was performed on an Attune NxT Flow Cytometer (Thermo Fisher, Waltham, MA) and analyzed using Attune Nxt software. Percent positive events were determined against samples stained with matching isotype controls.

### Cellspan esophageal implant preparation, culture, and transport

Pediatric sized scaffolds were electrospun, loaded into bioreactors and sterilized prior to cell seeding. Images of electrospun scaffolds of various sizes, scanning electron microscopy (SEM) images of the native scaffold fiber network as well as cell attachment are presented in Supplementary Fig. 4. Prior to seeding, cells were expanded out to at least the second passage. Seven days prior to surgery, cells were seeded onto a 12 mm ID × 100 mm L Cellframe™ scaffold at a density of ~4000 cells/mm^2^ for each implant (Supplementary Fig. 5a). In addition, for every lot of CEIs scheduled for implant, an additional sentinel CEI was prepared for Quality Control (QC) release assays. CEIs were not produced for surgical control animals. CEIs were incubated (37 °C, 5% CO_2_) in a custom rotating bioreactor for 6 days with media exchanges every 48 h until harvest. Upon harvest, QC assays were performed on the sentinel CEI as well as sterility assays on the implant CEI condition media. The implant CEIs were placed in shipping tubes with pre-gassed alpha MEM + HEPES (Gibco, Grand Island, NY) + 1% gentamicin (Gibco, Grand Island, NY) and cooled to 4 °C until they were transferred to pre-cooled shipping boxes. CEIs were shipped by courier to surgical sites with temperature monitors (2–8 °C).

### Viability and metabolic activity of cellspan esophageal implant

Viability of cells on QC sentinel CEIs was performed as previously described^32^. Glucose and lactate concentrations were measured in fresh medium and medium collected on days 2, 4, and 6 from all CEIs using a Nova Stat Profile Prime analyzer (Nova Biomedical; Waltham, MA) (Supplementary Fig. 5b).

### Cell Dose and cell penetration on the cellspan esophageal implant scaffold

Cell viability and cell penetration was determined on dedicated QC sentinel CEIs as previously described^32^, (Supplementary Fig. 5d). To determine cell numbers on scaffold, five punches were taken from along the length of QC CEIs and frozen at −80 °C. Frozen punch samples were thawed and then lysed using an SDS based buffer + proteinase K (Blood and Tissue DNA Extraction Kit, Qiagen, Germantown, MD) and subsequently homogenized using a bead mill (Thermo Fisher Scientific, Waltham, MA). DNA was extracted from lysates according to the manufacturer’s instructions (Qiagen, Germantown, MD). DNA was quantified via QuBit, using DNA BR Qubit assay kit (Thermo Fisher Scientific, Waltham, MA). Cell number was calculated by dividing DNA content by 5.1 pg DNA/diploid porcine cell and dividing by the area of a punch. Cell number on the QC was extrapolated from DNA content and calculated as cells per mm^2^. (Supplementary Fig. 5e).

### Cellspan esophageal implant surgery and initial stent placement

All animal procedures were performed at American Preclinical Services (APS, Minneapolis, MN. Approved IACUC protocols OIP007-IS75 and OIP010-IS75). APS has the following certifications and accreditations: USDA registration number 41-R0074; AAALAC accreditation number 001236; and PHS assurance number A4586-01.

Piglets were placed under general anesthesia, prepped, and draped according to standard operating procedures. The piglet was placed in a supine position and a 5 cm transverse incision was made in a left subcostal position and the abdominal cavity was entered. The stomach was identified, and two purse-string sutures were placed. An incision was made lateral on the left to the incision site and a 14 Fr Mic tube (Kimberly Clark, Neenah, WI) was placed through the incision and tunneled to the stomach. The tube was filled with water, purse-strings secured, and the stomach was tacked to the underside of the abdominal wall. The fascia and skin were closed with absorbable sutures. Next, an oblique incision was made in the right neck and the jugular vein was isolated. The venous access port (VAP) (Kimberly Clark, Neenah, WI) was placed laterally in a subcutaneous pocket. The tubing was then tunneled to the area where the vein was accessed. The tubing was cut to size, a venotomy was made and the tubing was fed into the vein into a central position. A vicryl tie (Ethicon, Somerville, NJ) around the vein and catheter was secured to keep it in place and the port was secured subcutaneously. The Huber needle was left in place and secured, and the incision was closed with absorbable suture. Finally, a standard right posterolateral thoracotomy was performed between the fourth and fifth intercostal space. The thoracic cavity was entered, and the lung retracted medially. The esophagus was isolated, preserving the vagus nerve, and a 5 cm segment of esophagus was removed in animals receiving CEIs. A 4-0 PDS stay suture is placed proximally and distally in the esophagus above and below the area of transection. The esophagus was then transected. The seeded CEI was trimmed on each side to a 6 cm length and sewn to the proximal and distal esophageal ends. The back wall of the proximal esophagus is sewn with 4-0 PDS in a running fashion. An orogastric tube is passed into the lumen and the anastomosis is completed proximally and distally over the tube with a 4-0 PDS suture. For the surgical control animals, a 2 cm segment was removed, followed by an end-to-end anastomosis using 4-0 PDS in a running fashion. A 12 mm × 100 mm Alimaxx esophageal stent (Merit Medical, South Jordan, UT) was placed using direct vision and fluoroscopy. The ribs were re-approximated, and the muscles were closed in 2 layers with absorbable suture (Ethicon, Somerville, NJ). A chest tube was placed in the thoracic space during closure and when the muscles were re-approximated, air was evacuated from the chest by giving positive pressure breaths (Valsalva maneuver). Skin was re-approximated with absorbable suture. A summary of the procedure is found in Supplementary Fig. 2.

### Animal in-life management

Animals were housed together in raised pens leading up to the initial procedure. The animals were then housed individually in raised pens with normal food and water following the adipose biopsy and other subsequent procedures. Animals were fasted for 12–24 h prior to all procedures. Animal health, including evaluation of incision site and clinical observations, body weights/condition, endoscopy/fluoroscopy, barium swallows and clinical pathology, was monitored at regular intervals. Starting day one post-implant, animals were fed a liquid nutrient diet through the implanted G-tube and allowed access to water only. Animals were slowly transitioned to a soft oral diet starting at ~21 days after implantation. Cohort 1 animals (365 ± 15 days) were transitioned to solid food starting at ~Day 35 after CEI implant. Cohort 2 (90 ± 3 days) and Cohort 3 (30 ± 3 days) maintained liquid oral diet through termination (~Day 90 and ~Day 30, respectively).

### Endoscopic assessments and stent exchanges

Animals were prepped and sedated per facility standard operating procedures (SOPs). Fully covered nitinol stents manufactured by Merit Medical (South Jordan, UT) and Boston Scientific (Marlborough, MA) were exchanged as necessary up to a 23 mm diameter stent. The initial stents were deployed immediately after surgery and subsequently removed at day 21 post-implantation (Fig. 1). To accommodate for growth of the animal, the stents were exchanged every 3–4 weeks with increasing diameter sizes unless symptoms of migration or obstructions presented sooner. To visualize the esophageal stent, an endoscope with live video feed was used in conjunction with fluoroscopy. If the esophageal stent was removed, the esophagus was visualized after removal to determine if the esophagus sustained any injury during the process as well as to monitor regeneration (Fig. 2). A new stent was then inserted and deployed under fluoroscopic and endoscopic guidance. For Cohort 1, stents were permanently discontinued once the mucosal layer was fully formed and the maximum stent diameter (23 mm) was reached (between 3 and 6 months post-implant). For Cohort 2 and Cohort 3, the stents were utilized until study termination. A summary of endoscopic interventions for each cohort can be found in Table 2.

### Evaluation of tissue regeneration by CT scan

Post-operative (Post-Op) CT imaging was performed on animals in all 3 cohorts (30, 90, and 365 days) at various time points, as listed in Supplementary Table 2. The esophageal stents were in place during the scanning process for the short-term assessments (up to day 90), however, the stents were removed (as per protocol) 3–6 months post implantation and therefore the CT imaging at 365 days was performed in the absence of esophageal stents. The scan recordings, DICOM image files, were analyzed by an independent medical imaging core laboratory (Medical Metrics, Inc., Houston, TX) for retrospective analysis. The CEI implant zone was determined for the first available post-op time point by identifying the anastomosis sites as surrogates for the CEI boundaries, then deriving the CEI Central Slice as the superior-inferior midpoint between the anastomosis sites. Quantitative measurements were obtained by an analyst. The analyst and radiologist were not blinded to the treatment group or time points for each subject. Quantitative measurements and qualitative assessments required identification of the CEI Implant Zone. Because the CEI was radiolucent, alternative anatomical features were used including the anastomosis sites and the carina, which were identified by the analyst (Fig. 3).

### Barium swallow esophagram

Animals were brought to a procedure room, placed in a sling or on a table and fed an oral mixture of food with barium while recording fluoroscopic video at various timepoints during the study. Static images were obtained to demonstrate patency of the construct and ability of barium to traverse from the mouth to the stomach (Fig. 4).

### Histopathology and immunohistochemistry

At experiment termination, the esophagus and designated representative tissues were collected and processed for histomorphology. Samples were processed by American Preclinical Services, LLC (APS). All slides were stained with hematoxylin and eosin (H&E) and with Masson’s Trichrome (MT). Slides were sent to StageBio, Inc., (Mt Jackson, VA) for imaging, histopathology review, immunohistochemical analysis, scoring and reporting. Immunohistochemical analysis was performed after deparaffinization with xylene. Antigen retrieval was performed using 0.015% citraconic anhydride at 95 °C for 15 min followed by blocking of endogenous peroxidase activity. Primary antibody was incubated at room temperature for 1 h to identify marker CK13, smooth muscle marker SM22, and axonal growth marker GAP43. Slides were then incubated with secondary antibody for 1 h at room temperature, developed using DAB and counterstained with hematoxylin. Antibodies used for immunohistochemical analysis are included in Supplementary Table 1.

### Reporting summary

Further information on research design is available in the Nature Research Reporting Summary linked to this article.

## Supplementary information

Supplementary Figures and Tables

Reporting summary

## Data Availability

All non-proprietary datasets generated during and/or analyzed during the current study are available from the Biostage, Inc., on reasonable request.
