# Supplementary material for: Esophageal regeneration following surgical implantation of a tissue engineered esophageal implant in a pediatric model
Source: NPJ Regen Med. 2022 Jan 10;7:1. doi: 10.1038/s41536-021-00200-9 (PMC8748753; doi:10.1038/s41536-021-00200-9)
Supplement: Supplementary file 1 — Supplementary Figures and Tables [file 41536_2021_200_MOESM1_ESM.pdf]

Supplementary Figures and Tables

Supplementary Figure 1. Types and classification of Esophageal atresia

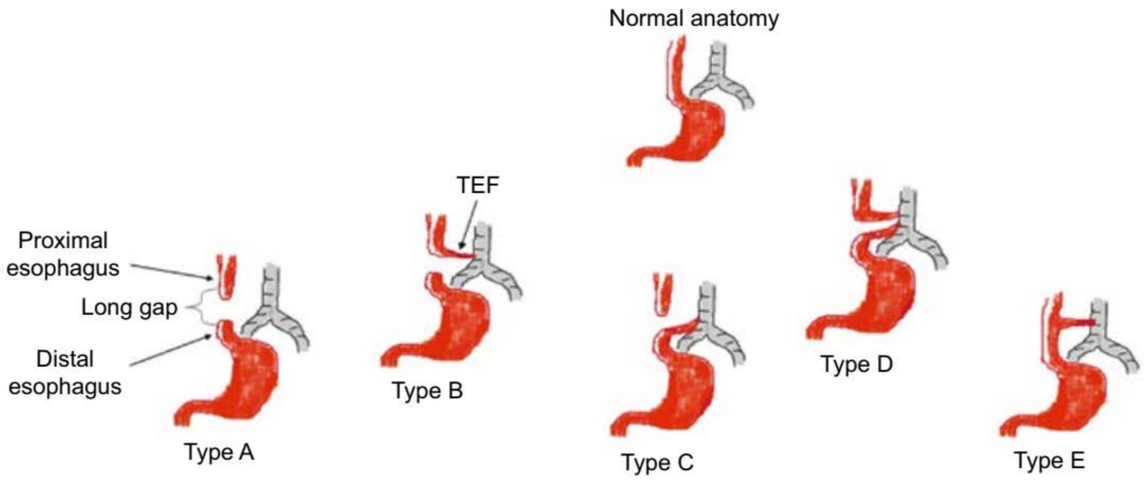

Long gap esophageal atresia presents with two blind ends of the esophagus and a missing esophageal segment (Gross type A, Vogt type II). EA Gross type B is defined as EA with a proximal fistula (Vogt type IIIA). EA with distal fistula is classified as Gross type C (Vogt IIIB) and EA with double fistulae is classified as Gross type D (Vogt IIIC) and Gross type E (H-type).

Supplementary Figure 2. Surgical Paradigm

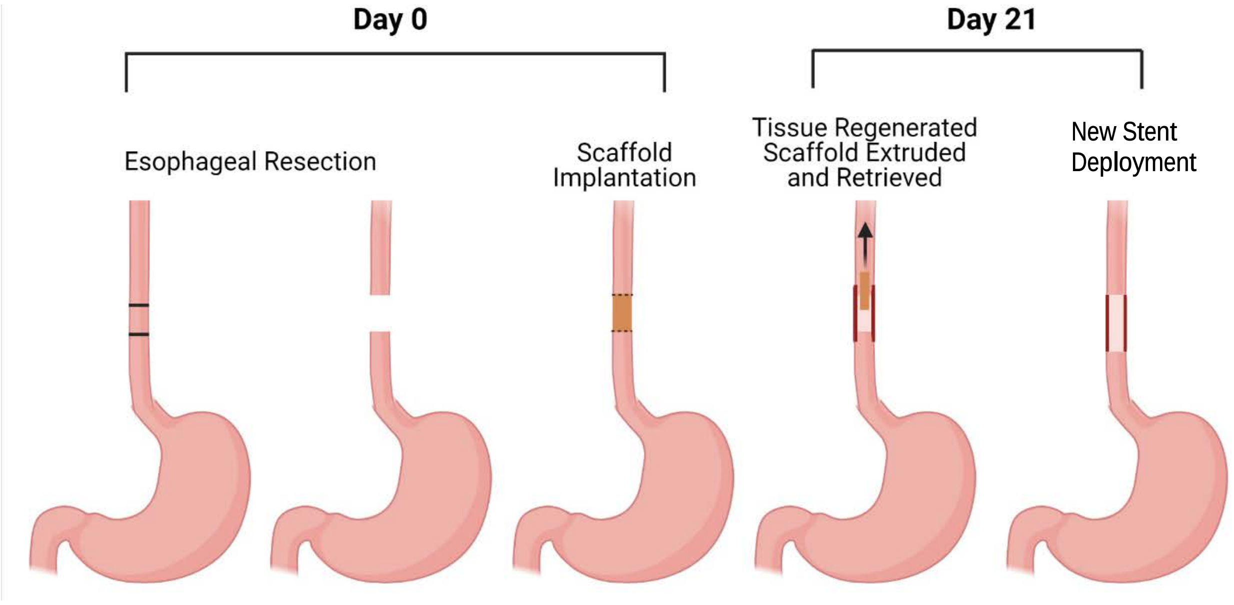

1 2 3 4 5

Resection and Implantation Steps

Following implantation (Steps 1-3) an initial stent is deployed to maintain lumenal patency (Step 3). By day 21 post-implantation, the initial stent is removed along with the scaffold component revealing a patent fibrovascular tube of tissue (Step 4). A second stent is deployed (Step 5) and typically is retained for the following 3-6 months until further healing of the tissue permits final stent removal.

Supplementary Figure 3. pAd-MSC Phenotypic Characterization

a

Cell Line	PDT (P1-P2)	Viability
18P0973	19.4 h	92 %
18P0974	20.8 h	94 %
19P0367	21.2 h	94 %
19P0368	20.9 h	96 %
18P0633	23.6 h	78 %
18P0634	24.9 h	98 %
18P0635	21.4 h	93 %
18P0977	18.5 h	83 %
18P0975	26.3 h	92 %
18P0459	21.9 h	85 %
18P0460	22.7 h	78 %
18P0461	22.2 h	94 %

b

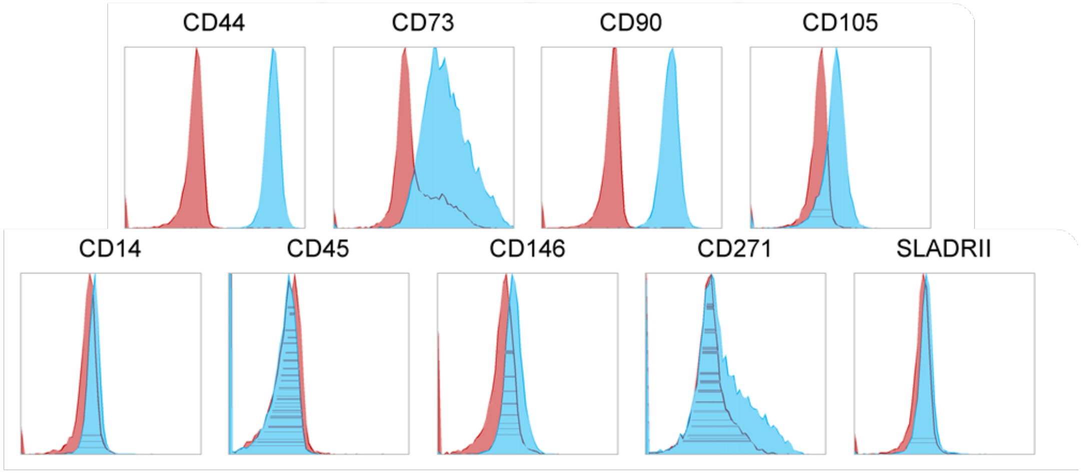

c

Cell Line	CD44	CD90	CD105	CD73	CD14	CD45	CD146	CD271	SLADRII
18P0973	99.9	99.9	91.4	83.0	0.9	0.5	2.8	3.6	4.3
18P0974	99.8	100.0	90.1	55.8	3.9	0.8	6.4	4.3	3.9
19P0367	100.0	99.9	60.4	47.0	0.2	1.1	0.5	1.6	0.2
19P0368	100.0	99.8	88.6	79.6	0.2	1.1	5.0	2.9	1.7
18P0633	98.5	76.2	91.4	90.2	NA	1.2	14.7	5.1	1.8
18P0634	99.6	68.3	89.8	93.9	NA	1.1	27.0	5.2	3.7
18P0635	98.5	82.9	93.8	94.0	NA	2.8	23.7	11.5	5.7
18P0977	99.9	98.7	NA	99.4	87.5	2.7	20.4	1.1	0.2
18P0975	99.9	73.1	82.5	99.9	4.8	0.5	26.4	12.1	2.3
18P0459	99.9	95.2	56.1	NA	3.3	0.2	11.8	0.8	0.7
18P0460	99.7	79.6	35.2	NA	3.0	0.1	8.0	0.3	0.8
18P0461	99.9	84.5	89.2	NA	7.9	0.1	61.2	0.6	0.5

(**a**) Table of population doubling time calculated for pAd-MSCs between passage 1 and passage 2 and viability at P2, determined via cell count using trypan blue. Calculations based upon live cells only. (**b**) Representative flow cytometry peaks of pAd-MSCs (18P0975, Passage 2). (**c**) Percent positive cells of each indicated cell surface marker as determine by flow cytometry.

Supplementary Figure 4. Scaffold Design and CEI Final Product

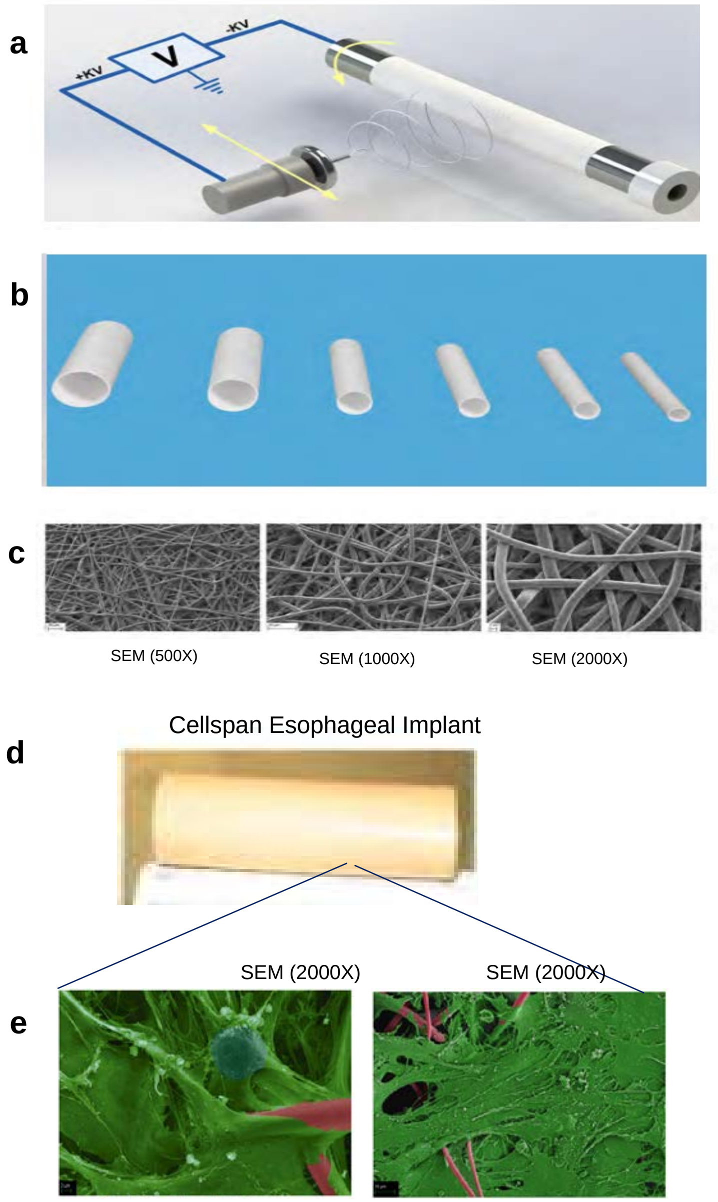

Electrospinning apparatus and scaffold mandril (**a**). Various sized tubular scaffolds (**b**). Scanning electron micrographs of the scaffold overlapping fibers (500x, 1000x and 2000x magnifications, left to right panels, respectively (**c**). CEI final cell seeded product-post bioreactor incubation (**d**). Colorized SEM micrographs of cells (green) attached to scaffold fibers (red, **e**).

Supplementary Figure 5. CEI Characterization

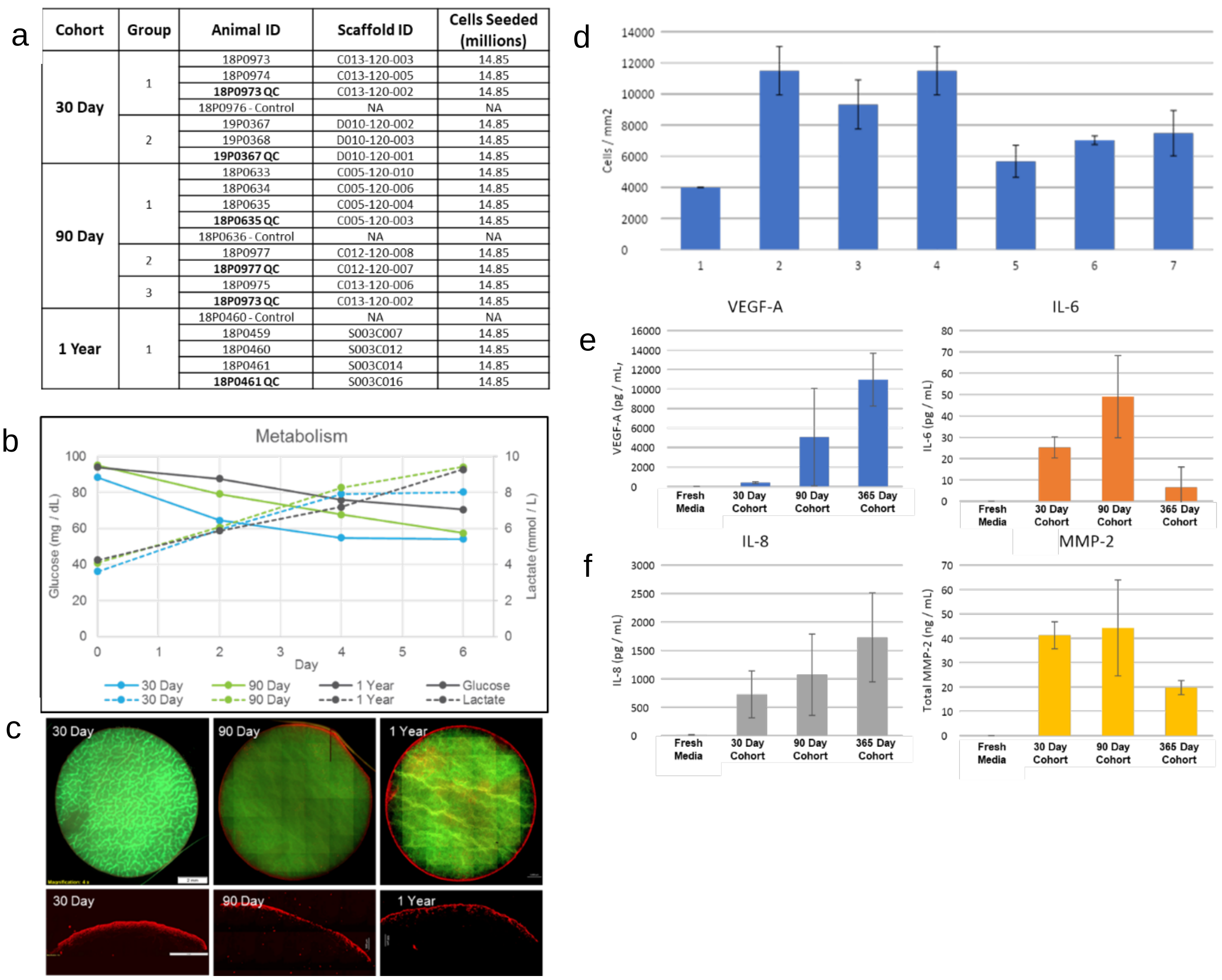

Cohort	Group	Animal ID	Scaffold ID	Cells Seeded (millions)
30 Day	1	18P0973	C013-120-003	14.85
		18P0974	C013-120-005	14.85
		**18P0973 QC**	C013-120-002	14.85
		18P0976 - Control	NA	NA
	2	19P0367	D010-120-002	14.85
		19P0368	D010-120-003	14.85
		**19P0367 QC**	D010-120-001	14.85
90 Day	1	18P0633	C005-120-010	14.85
		18P0634	C005-120-006	14.85
		18P0635	C005-120-004	14.85
		**18P0635 QC**	C005-120-003	14.85
		18P0636 - Control	NA	NA
	2	18P0977	C012-120-008	14.85
		**18P0977 QC**	C012-120-007	14.85
	3	18P0975	C013-120-006	14.85
		**18P0973 QC**	C013-120-002	14.85
1 Year	1	18P0460 - Control	NA	NA
		18P0459	S003C007	14.85
		18P0460	S003C012	14.85
		18P0461	S003C014	14.85
		**18P0461 QC**	S003C016	14.85

Metabolic and viability. (A) Table of CEI production and cell seeding number information. (B). Average metabolic readings for each cohort of CEIs. Glucose values are represented by solid lines, while lactate values, plotted on the secondary axis, are represented by dashed lines. (C). Representative images of cell viability on quality control CEIs (30 day QC, 90 Day QC, 1 year QC). QC CEIs were analyzed using a LIVE/DEAD assay where CEI punches were stained with calcein AM (green; live cells) and ethidium bromide (red; dead cells). (D) Representative images of translocation of cells throughout quality control CEIs (30 day QC, 90 Day QC, 1 year QC). Fixed CEIs were cryosectioned and stained with ethidium bromide (red; fixed cells) to visualize the depth of cellular penetration. (E) Cell quantification of QC CEIs. Punches from each QC CEI were frozen for >24 hours, then DNA was extracted and quantified. Cell number per QC punch was extrapolated from DNA quantity. (F) Cytokine expression of CEIs in each cohort. Conditioned media was tested for the indicated cytokines via ELISA. Averages from 5 punch samples are displayed for each cohort. Error bars represent the SEM from the 5 samples.

Supplementary Figure 6. Growth Chart for 365-day animals

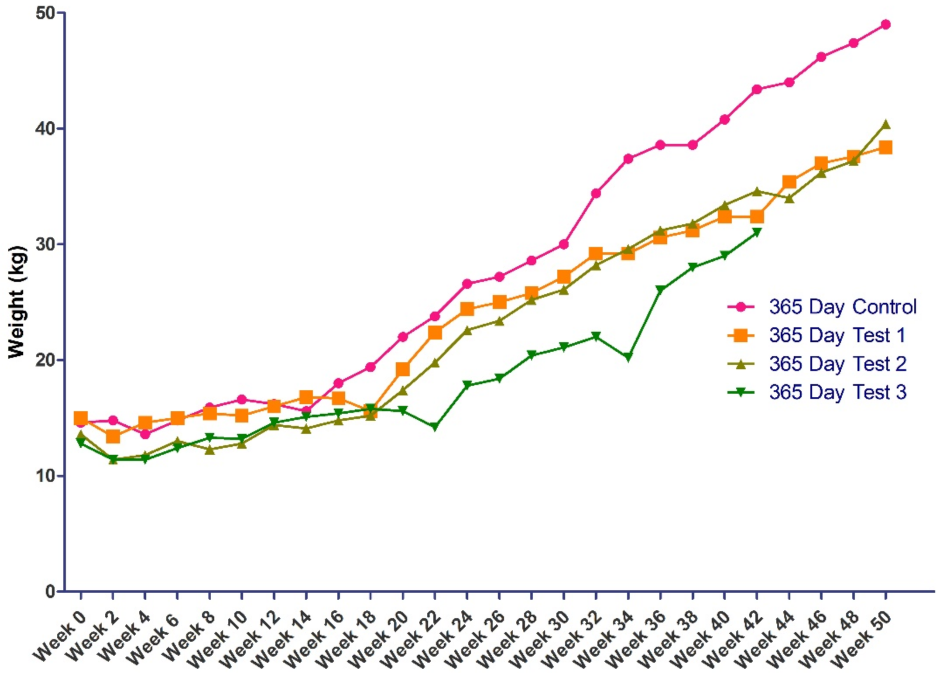

Supplementary Figure 7. Diagram of the various tissue zones in both treated and control animals.

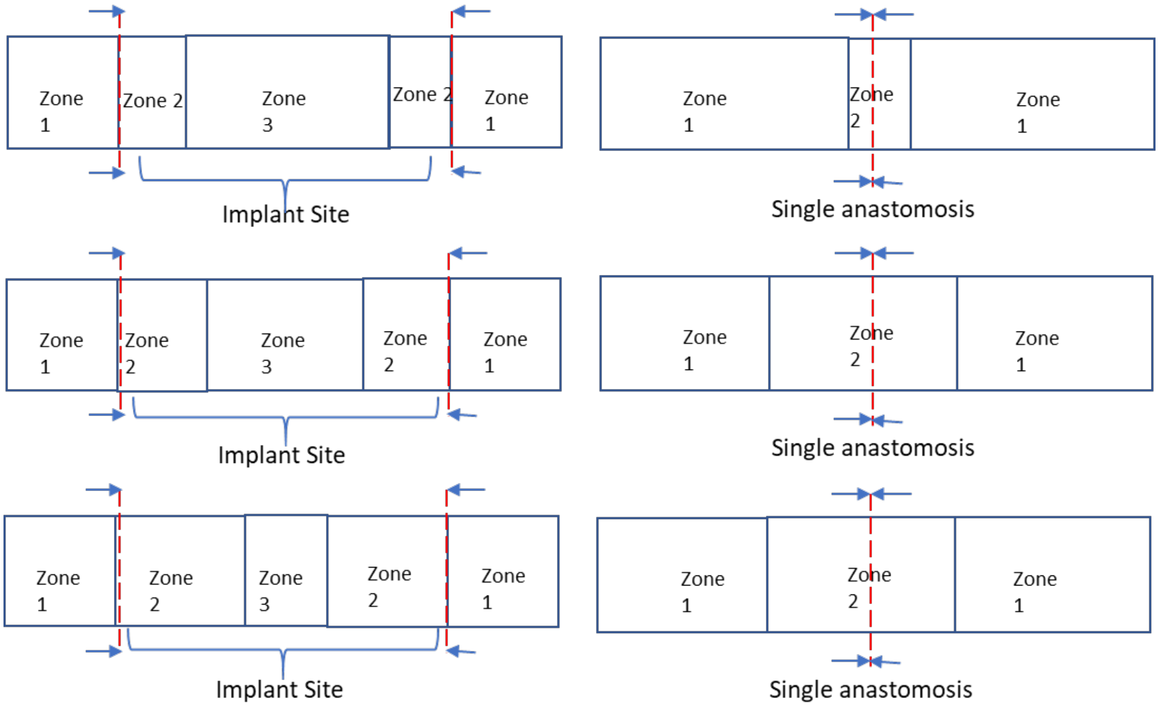

Supplementary Table 1. Flow Cytometry & Primary Antibodies

Antibody	Color	Company	Catalog #
CD105	PerCP	Novus	NB110-58718PCP
CD14	AF488	Novus	FAB4597G
CD146	FITC	BioRad	MCA2141F
CD271	Unconjugated	Thermo Fisher	12-9400-82
CD44	PECy7	Thermo Fisher	25-0441-82
CD45	PECy7	Thermo Fisher	25-0459-42
CD73	Unconjugated	RD Systems	AF4488
CD90	PECy7	Thermo Fisher	25-909-42
CK13	Unconjugated	Invitrogen	MA1-90939
GAP43	Unconjugated	Novus Biologics	NB300-143SS
Isotype Control	FITC	Thermo Fisher	11-4732-42
Isotype Control	FITC	Thermo Fisher	11-4714-82
Isotype Control	PECy7	Thermo Fisher	25-4714-42
Isotype Control	PECy7	Thermo Fisher	25-4732-81
Isotype Control	PerCP	Biolegend	400250
Isotype Control	Unconjugated	Thermo Fisher	31243
Isotype Control	Unconjugated	Thermo Fisher	14-4714-82
Secondary Antibody	FITC	Thermo Fisher	31627
Secondary Antibody	PECy7	Biolegend	406614
Secondary Antibody-Mouse	HRP	Biocare	BRR4002
Secondary Antibody-Rabbit	HRP	Biocare	BRR4009
SLADRII	FITC	BioRad	MCA2314F
SM22	Unconjugated	Abcam	Ab14106

Supplementary Table 2. CT Imaging Timepoints

Animal ID	Cohort	Device Group	Timepoint
18P0458	1-365 Day	Control	Day 350
18P0459	1-365 Day	Test	Day 118
18P0459	1-365 Day	Test	Day 297
18P0460	1-365 Day	Test	Day 349
18P0461	1-365 Day	Test	Day 350
18P0975	2- 90 Day	Test	Day 0
18P0975	2- 90 Day	Test	Day 7
18P0975	2- 90 Day	Test	Day 20
18P0975	2- 90 Day	Test	Day 90
18P0976	3- 30 Day	Control	Day 7
18P0976	3- 30 Day	Control	Day 20
18P0973	3- 30 Day	Test	Day 7
18P0973	3- 30 Day	Test	Day 21
18P0974	3- 30 Day	Test	Day 20

Supplementary Table 3. General Histopathologic Changes at Day 30 post implantation

ANATOMIC REGION	Day 30 Test<br>*Animal 18P0973 (slides 2,3,6,7); Animal 19P0368 (slides 2,5); Animal 18P0974 (slides 2,5); Animal 19P0367 (slides 2,5)*			Day 30 Control (Zone 2 only)<br>*Animal 19P0976 (slides 2,5)*
	**ZONE 1**	**ZONE 2**	**ZONE 3**	**ZONE 2**
Mucosa<br>(Epithelium, Lamina Propria, Muscularis Mucosae)	*Ulceration in 1/4 animals. Epithelial hyperplasia in 4/4 animals.*	*Epithelialization complete with epithelial hyperplasia in 4/4 animals.<br>Lamina propria: minimal to moderate fibrovascular tissue and minimal to moderate inflammation. Muscularis mucosae: Mixed areas of muscular and fibrovascular tissue. Minimal to mild inflammation.*	*Minimal epithelialization with epithelial hyperplasia in 3/4 animals. Remaining surface is not epithelialized. Fragments of scaffold-like material present on the mucosal surface of 1/4 animals (Animal 19P0368). No evidence of smooth muscle fibers.*	*Minimal epithelialization with epithelial hyperplasia. Remaining surface is markedly unepithelialized.*
Submucosa	*Mostly normal.*	*Minimal to moderate fibrovascular tissue and inflammation.*	*Moderate to marked fibrovascular tissue replaces submucosa in 4/4 animals. Normal submucosa is not present.*	*Marked fibrovascular tissue replaces submucosa. Normal submucosa is not present.*
Tunica Muscularis Propria	*Normal*	*Minimal, mild or moderate amounts of muscle in 4/4 animals. Minimal to moderate fibrovascular tissue and inflammation. Neovascularization not detected.*	*Moderate to marked fibrovascular tissue replaces tunica muscularis in 4/4 animals. Normal tunica muscularis is not present. Neovascularization not detected.*	*Moderate to marked fibrovascular tissue replaces tunica muscularis. Normal tunica muscularis is not present. Neovascularization not detected.*
Adventitia	*Minimal to marked fibrovascular tissue in 4/4 animals. Lung adhesions in 1/4 animals.*	*Mild to marked fibrovascular tissue in 4/4 animals. Lung adhesions in 1/4 animals.*	*Mild to marked fibrovascular tissue in 4/4 animals. Lung adhesions in 2/4 animals.*	*Moderate to marked fibrovascular tissue. Lung adhesions present.*

Changes may vary between sections and animals. In Zone 3 (Test) and Zone 2 (Control), a comment on neovascularization was added to the Tunica Muscularis Propria line for organization purposes but there was no differentiation between normal esophageal layers in these areas.

Supplementary Table 4. General Histopathologic Changes at Day 90 post implantation

General Histopathologic Changes Typical of Each Zone in Esophageal Tissue Sections at Day 90				
**ANATOMIC REGION**	**Day 90 Test** *Animal 18P0633 (slides 2,5); Animal 18P0634 (slides 2,5); Animal 18P0975 (slides 17,20)*			**Day 90 Control (Zone 2 only)** *Animal 18P0636 (slides 2,5)*
	**ZONE 1**	**ZONE 2**	**ZONE 3**	**ZONE 2**
Mucosa (Epithelium, Lamina Propria, Muscularis Mucosae)	*Epithelial hyperplasia in 3/3 animals.*	*Epithelialization complete with epithelial hyperplasia in 3/3 animals. Lamina propria: mild to moderate fibrovascular tissue and minimal to mild inflammation. Muscularis mucosae: Mixed areas of muscular and fibrovascular tissue. Minimal to mild inflammation.*	*Epithelialization complete or nearly complete with epithelial hyperplasia in 2/2 animals assessed. Epithelialization not assessed in 1/3 animals. Mild absence of epithelialization in 1/3 animals. Fibrovascular tissue replaces lamina propria and muscularis mucosae, which are not present. Scattered smooth muscle fibers in 3/3 animals.*	*Epithelialization complete with epithelial hyperplasia. Scattered smooth muscle fibers.*
Submucosa	*Normal*	*Mild to moderate fibrovascular tissue and minimal to mild inflammation.*	*Marked fibrovascular tissue replaces submucosa. Normal submucosa is not present.*	*Marked fibrovascular tissue replaces submucosa. Normal submucosa is not present.*
Tunica Muscularis Propria	*Normal*	*Minimal amounts of muscle in 1/3 animals. Mild to moderate fibrovascular tissue and minimal to mild inflammation. Minimal neovascularization present in 3/3 animals.*	*Moderate fibrovascular tissue replaces tunica muscularis. Normal tunica muscularis is not present. Minimal neovascularization present in 3/3 animals.*	*Moderate to marked fibrovascular tissue replaces tunica muscularis. Normal tunica muscularis is not present. Minimal neovascularization present.*
Adventitia	*Minimal to moderate fibrovascular tissue in 2/3 animals. Lung adhesions in 1/3 animals.*	*Minimal to moderate fibrovascular tissue in 3/3 animals. Lung adhesions in 1/3 animals.*	*Minimal to moderate fibrovascular tissue in 3/3 animals. Lung adhesions in 2/3 animals.*	*Moderate fibrovascular tissue.*

Changes may vary between sections and animals. In Zone 3 (Test) and Zone 2 (Control), a comment on neovascularization was added to the Tunica Muscularis Propria line for organization purposes but there was no differentiation between normal esophageal layers in these areas.

Supplementary Table 5. General Histopathologic Changes at Day 365 post implantation

General Histopathologic Changes Typical of Each Zone in Esophageal Tissue Sections at Day 365				
**ANATOMIC REGION**	**Day 365 Test**<br>*Animal 18P0460 (slides 2,5); Animal 18P0461 (slides 2,5)*			**Day 365 Control (Zone 2 only)**<br>*Animal 18P0458 (slides 2,5)*
	**ZONE 1**	**ZONE 2**	**ZONE 3**	**ZONE 2**
Mucosa<br>(Epithelium, Lamina Propria, Muscularis Mucosae)	*Epithelial hyperplasia in 2/2 animals.*	*Epithelialization complete with epithelial hyperplasia in 2/2 animals. Lamina propria: mild to moderate fibrovascular tissue and minimal to mild inflammation. Muscularis mucosae: Mixed areas of muscular and fibrovascular tissue. Minimal to mild inflammation.*	*Epithelialization complete with epithelial hyperplasia in 2/2 animals. Fibrovascular tissue replaces lamina propria and muscularis mucosae, which are not present. Smooth muscle fibers in 2/2 animals.*	*Epithelialization complete with epithelial hyperplasia. Smooth muscle fibers present.*
Submucosa	*Normal*	*Mild to moderate fibrovascular tissue and minimal to mild inflammation.*	*Moderate fibrovascular tissue and minimal to mild inflammation.*	*Moderate fibrovascular tissue and minimal inflammation.*
Tunica Muscularis Propria	*Normal*	*Mild to moderate fibrovascular tissue replaces tunica muscularis. Normal tunica muscularis not present. Minimal neovascularization present in ½ animals.*	*Moderate fibrovascular tissue replaces tunica muscularis. Normal tunica muscularis not present. Minimal vascularization present in ½ animals and mild vascularization present in ½ animals.*	*Moderate fibrovascular tissue replaces tunica muscularis. Normal tunica muscularis not present. Mild vascularization present.*
Adventitia	*Minimal to moderate fibrovascular tissue in 2/2 animals.*	*Minimal to moderate fibrovascular tissue in 2/2 animals.*	*Mild to moderate fibrovascular tissue in 2/2 animals. Lung adhesions in 2/2 animals.*	*Moderate fibrovascular tissue.*

Changes may vary between sections and animals. In Zone 3 (Test) and Zone 2 (Control), a comment on neovascularization was added to the Tunica Muscularis Propria line for organization purposes but there was no differentiation between normal esophageal layers in these zones.

Supplementary Table 6. Immunohistochemical Findings at Day 365 post implantation

<table>
<tr><th rowspan="2">ANATOMIC REGION</th><th colspan="3">Day 365 Test
Animal 18P0460 (slide 2);
Animal 18P0461 (slide 2)</th><th>Day 365 Control
(Zone 2 only)
Animal 18P0458 (slide 2)</th></tr>
<tr><th>ZONE 1</th><th>ZONE 2</th><th>ZONE 3</th><th>ZONE 2</th></tr>
<tr><td>Epithelium</td><td colspan="3">*Suprabasal epithelium: CK13, CK14.*
Basal epithelium: CK14, p63, Ki67.
Inflammatory cells: Ki67.</td><td>*Suprabasal epithelium: CK13, CK14.*
Basal epithelium: CK14, p63.</td></tr>
<tr><td>Lamina Propria</td><td colspan="2">*Blood vessels: SMA and SM22 (smooth muscle), CD31 (endothelium).*
Nerve fibers: GAP-43.
Inflammatory cells: Ki67.</td><td rowspan="5">*No discernible layers. All structures present in fibrovascular tissue.*
Blood vessels: SMA and SM22 (smooth muscle), CD31 (endothelium).
Myofibroblasts: SMA and SM22.
Scattered smooth muscle fibers: SMA and SM22.
Nerve fibers: GAP-43.
Inflammatory cells: Ki67.</td><td rowspan="5">*No discernible layers. All structures present in fibrovascular tissue.*
Blood vessels: SMA and SM22 (smooth muscle), CD31 (endothelium).
Myofibroblasts: SMA and SM22.
Scattered smooth muscle fibers: SMA and SM22.
Nerve fibers: GAP-43.
Inflammatory cells: Ki67.</td></tr>
<tr><td>Muscularis Mucosae</td><td colspan="2">*Smooth muscle: SMA and SM22.*
Blood vessels: SMA and SM22 (smooth muscle), CD31 (endothelium).
Nerve fibers: GAP-43.
Inflammatory cells: Ki67.</td></tr>
<tr><td>Submucosa</td><td colspan="2">*Blood vessels: SMA and SM22 (smooth muscle), CD31 (endothelium).*
Nerve fibers: GAP-43.
Inflammatory cells: Ki67.</td></tr>
<tr><td>Tunica Muscularis Propria</td><td colspan="2">*Striated skeletal muscle: MYL1 (MY32).*
Smooth muscle: SMA and SM22.
Blood vessels: SMA and SM22 (smooth muscle), CD31 (endothelium).
Nerve fibers: GAP-43.
Inflammatory cells: Ki67.</td></tr>
<tr><td>Adventitia</td><td colspan="2">*Blood vessels: SMA and SM22 (smooth muscle), CD31 (endothelium).*
Nerve fibers: GAP-43.
Inflammatory cells: Ki67.</td></tr>
</table>
