# Supplementary material for: Esophageal regeneration following surgical implantation of a tissue engineered esophageal implant in a pediatric model
Source: NPJ Regen Med. 2022 Jan 10;7:1. doi: 10.1038/s41536-021-00200-9 (PMC8748753; doi:10.1038/s41536-021-00200-9)
Supplement: Supplementary file 2 — Reporting summary [file 41536_2021_200_MOESM2_ESM.pdf]

nature research

Corresponding author(s): William Fodor

[ ] Initial submission [X] Revised version [ ] Final submission

# Life Sciences Reporting Summary

Nature Research wishes to improve the reproducibility of the work that we publish. This form is intended for publication with all accepted life science papers and provides structure for consistency and transparency in reporting. Every life science submission will use this form; some list items might not apply to an individual manuscript, but all fields must be completed for clarity.

For further information on the points included in this form, see Reporting Life Sciences Research. For further information on Nature Research policies, including our data availability policy, see Authors & Referees and the Editorial Policy Checklist.

## ▸ Experimental design

### 1. Sample size

Describe how sample size was determined.

Test animal cohorts (N=3) for each time point was determined to be the minimum number of animals to show reproducibility of the results. Attrition was accounted for since the test requires chest surgery. It was also determined that a single animal for the surgical control group was sufficient to control for the surgical procedure for each time point

### 2. Data exclusions

Describe any data exclusions.

Animals that died or were euthanized prior to the scheduled time point were excluded from the histology data

### 3. Replication

Describe whether the experimental findings were reliably reproduced.

Each experiment presented in the paper was repeated in multiple animals (between 3 and 5). While a few animals were excluded from analysis due to early termination, we observed consistent resiults between animals within the same cohorts. All of this information is presented in the text and in the tabulated summaries.

4. Randomization

Describe how samples/organisms/participants were allocated into experimental groups.

Animals were assigned sequentially in a random fashion to experimental and control groups.

5. Blinding

Describe whether the investigators were blinded to group allocation during data collection and/or analysis.

The investigators were not blinded during data collection. Histopathology was performed by an independent contract research organization

Note: all studies involving animals and/or human research participants must disclose whether blinding and randomization were used.

6. Statistical parameters

For all figures and tables that use statistical methods, confirm that the following items are present in relevant figure legends (or in the Methods section if additional space is needed).

| n/a | Confirmed | |
| --- | --- | --- |
| ☐ | ☒ | The exact sample size ($n$) for each experimental group/condition, given as a discrete number and unit of measurement (animals, litters, cultures, etc.) |
| ☐ | ☒ | A description of how samples were collected, noting whether measurements were taken from distinct samples or whether the same sample was measured repeatedly |
| ☐ | ☒ | A statement indicating how many times each experiment was replicated |
| ☐ | ☒ | The statistical test(s) used and whether they are one- or two-sided (note: only common tests should be described solely by name; more complex techniques should be described in the Methods section) |
| ☒ | ☐ | A description of any assumptions or corrections, such as an adjustment for multiple comparisons |
| ☒ | ☐ | The test results (e.g. $P$ values) given as exact values whenever possible and with confidence intervals noted |
| ☐ | ☒ | A clear description of statistics including central tendency (e.g. median, mean) and variation (e.g. standard deviation, interquartile range) |
| ☐ | ☒ | Clearly defined error bars |

*See the web collection on statistics for biologists for further resources and guidance.*

## Software

Policy information about availability of computer code

7. Software

Describe the software used to analyze the data in this study.

Average values presented for cytokine data from conditioned media were obtained using microsoft excel

For manuscripts utilizing custom algorithms or software that are central to the paper but not yet described in the published literature, software must be made available to editors and reviewers upon request. We strongly encourage code deposition in a community repository (e.g. GitHub). *Nature Methods* guidance for providing algorithms and software for publication provides further information on this topic.

## Materials and reagents

Policy information about availability of materials

8. Materials availability

Indicate whether there are restrictions on availability of unique materials or if these materials are only available for distribution by a for-profit company.

Some restrictions will be placed on materials involved in this study.  All requests for Biostage proporiety materials must be provided in writing to Biostage, Inc.

9. Antibodies

Describe the antibodies used and how they were validated for use in the system under study (i.e. assay and species).

a list of antibodies used in this study is tabulated in the Supplemental information submitted with the manuscript.

10. Eukaryotic cell lines

| | |
|---|---|
| a. State the source of each eukaryotic cell line used. | primary porcine adipose derived mesenchymal stromal cells were isolated and cultured from biospy specimens. |
| b. Describe the method of cell line authentication used. | n/a |
| c. Report whether the cell lines were tested for mycoplasma contamination. | all cell cultures were tested for sterility and mycoplasma |
| d. If any of the cell lines used are listed in the database of commonly misidentified cell lines maintained by ICLAC, provide a scientific rationale for their use. | n/a |

## Animals and human research participants

Policy information about studies involving animals; when reporting animal research, follow the ARRIVE guidelines

11. Description of research animals

| | |
|---|---|
| Provide details on animals and/or animal-derived materials used in the study. | Yucatan mini-pigs were used for this study and purchased from a qualified vendor. All animal procedures were conducted at a certified CRO (AAALAC, USDA, PHS Assurance |

Policy information about studies involving human research participants

12. Description of human research participants

| | |
|---|---|
| Describe the covariate-relevant population characteristics of the human research participants. | Study did not involve human research participants. |
